# Genistein Protects Against Lead-Induced Cognitive Impairment Through a Glutathione-Dependent Redox–Mitochondrial Apoptosis Axis

**DOI:** 10.3390/molecules31132251

**Published:** 2026-06-26

**Authors:** Zhongting Lv, Zeyu Ma, Yong Pang, Hao Wang, Jie Zhang

**Affiliations:** 1 College of Food Science and Engineering, Jilin University, Changchun 130062, China; 2 Advanced Medical Research Institute, Shandong University, Jinan 250012, China

**Keywords:** genistein, lead, neurotoxicity, glutathione metabolism

## Abstract

Lead exposure remains a pervasive environmental and public health threat, imposing a substantial burden of neurodevelopmental and cognitive dysfunction, yet safe mechanism-oriented interventions remain limited. Genistein, a soybean-derived isoflavone with antioxidant and neuroprotective potential, may counter heavy metal-induced neural injury; however, whether its efficacy is associated with redox–metabolic remodeling is unclear. Here, we evaluated genistein in lead-exposed C57BL/6J mice and lead-challenged HT22 hippocampal neurons. Genistein improved novel-arm exploration and spatial memory without altering locomotor or swimming performance, and attenuated neuronal disorganization and apoptosis in hippocampal CA1, CA3 and dentate gyrus regions. These protective effects were accompanied by reduced blood and hippocampal lead accumulation, restored glutathione redox balance, enhanced antioxidant capacity, preserved mitochondrial integrity, and suppressed Bax/Caspase-3-associated apoptotic signaling. Importantly, because genistein also reduced hippocampal lead accumulation, the in vivo neuroprotection may reflect both reduced target-tissue lead burden and improved glutathione-related redox homeostasis. Untargeted metabolomics identified 59 genistein-responsive metabolites enriched mainly in glutathione metabolism, oxidative phosphorylation, and ascorbate/aldarate metabolism, linking metabolic remodeling to behavioral recovery and reduced oxidative-apoptotic injury. In HT22 cells, blockade of glutathione synthesis by buthionine sulfoximine markedly weakened genistein-mediated cytoprotection, mitochondrial membrane potential recovery, and apoptosis inhibition. Collectively, genistein mitigates lead-induced hippocampal neurotoxicity and cognitive impairment by restoring glutathione-centered redox–mitochondrial homeostasis, supporting its further development as a mechanistically defined dietary candidate for environmental pollutant-associated neural injury.

## 1. Introduction

Lead (Pb) exposure remains a persistent global environmental health challenge, and no level of exposure is considered completely safe [1]. As a cumulative toxic metal, lead can persist in the human body for decades and affect multiple organs, with the nervous system being among its most vulnerable targets, particularly in children [2]. Estimates cited by the World Health Organization indicate that lead exposure was attributable to more than 1.5 million deaths and over 33 million disability-adjusted life years worldwide in 2021. Although environmental source control and chelation-based clinical management are important for reducing exposure and body burden, these strategies have limited ability to reverse established neurological dysfunction once lead-induced pathological processes have been initiated [3,4]. This limitation highlights the need to identify safe and mechanism-oriented interventions that can target the biological events linking lead exposure to neuronal injury and cognitive impairment [5].

Lead-induced cognitive dysfunction arises from multiple interrelated pathological processes, among which oxidative stress, mitochondrial dysfunction, and neuronal death are considered central contributors [6,7]. Oxidative stress is not merely a downstream consequence of lead exposure but may act as a nodal event that converts metal burden into cellular injury [5,8]. Lead can disturb the pro-oxidant/antioxidant balance by promoting reactive oxygen species generation, impairing antioxidant enzyme activities, interacting with sulfhydryl-containing molecules, and weakening thiol-dependent redox-buffering systems. In the brain, glutathione is a major endogenous antioxidant that participates in peroxide detoxification, maintenance of protein thiol status, and protection of neural cells against oxidative injury [9]. Disruption of the reduced glutathione/oxidized glutathione (GSH/GSSG) balance may therefore compromise neuronal redox homeostasis and increase susceptibility to mitochondrial damage. Because mitochondria are both sources and targets of reactive oxygen species, sustained redox imbalance can impair mitochondrial integrity and activate mitochondria-dependent apoptotic signaling, including Bax/Bcl-2 imbalance and Caspase-3 cleavage. Owing to its high energy demand and critical role in learning and memory, the hippocampus is especially vulnerable to oxidative mitochondrial insults [10].

Natural products and food-derived bioactive compounds have attracted increasing attention as potential adjunctive strategies for mitigating environmental toxicant-induced neural injury [11,12]. Genistein, a soybean-derived isoflavone, has been widely studied for its antioxidant, anti-inflammatory, anti-apoptotic, and neuroprotective properties [13,14,15]. Preclinical evidence suggests that genistein can improve cognitive performance in several models of memory impairment, partly through reducing oxidative stress and neuroinflammation [16]. In the context of lead neurotoxicity, genistein has been reported to alleviate lead-induced spatial memory impairment and neuronal apoptosis through mechanisms involving nuclear factor erythroid 2-related factor 2 (Nrf2)-mediated antioxidant signaling and the mitogen-activated protein kinase (MAPK)/nuclear factor kappa B (NF-κB) signaling pathways [17]. However, although these findings support a protective role of genistein against lead-induced neuronal injury, the metabolic basis underlying this protection remains incompletely understood. In particular, whether genistein-mediated neuroprotection is accompanied by remodeling of brain redox metabolism, especially glutathione-related metabolic pathways, remains to be clarified.

Accordingly, this work aimed to determine whether genistein mitigates lead-evoked cognitive deficits and hippocampal neuronal damage, and to examine the association between redox–metabolic regulation and its neuroprotective effects. To address this question, we combined behavioral testing, hippocampal histopathology, measurements of lead burden and oxidative stress, mitochondrial ultrastructural analysis, apoptosis-related protein detection, untargeted metabolomics, correlation analysis, and in vitro cellular experiments. This integrated approach was designed to determine whether genistein-mediated neuroprotection is associated with redox–metabolic remodeling and to provide further insight into the relationship among glutathione homeostasis, mitochondrial preservation, and neuronal apoptosis after lead exposure.

## 2. Results

### 2.1. Genistein Attenuates Lead-Evoked Cognitive Deficits Without Altering Motor Activity

**Figure 1:**
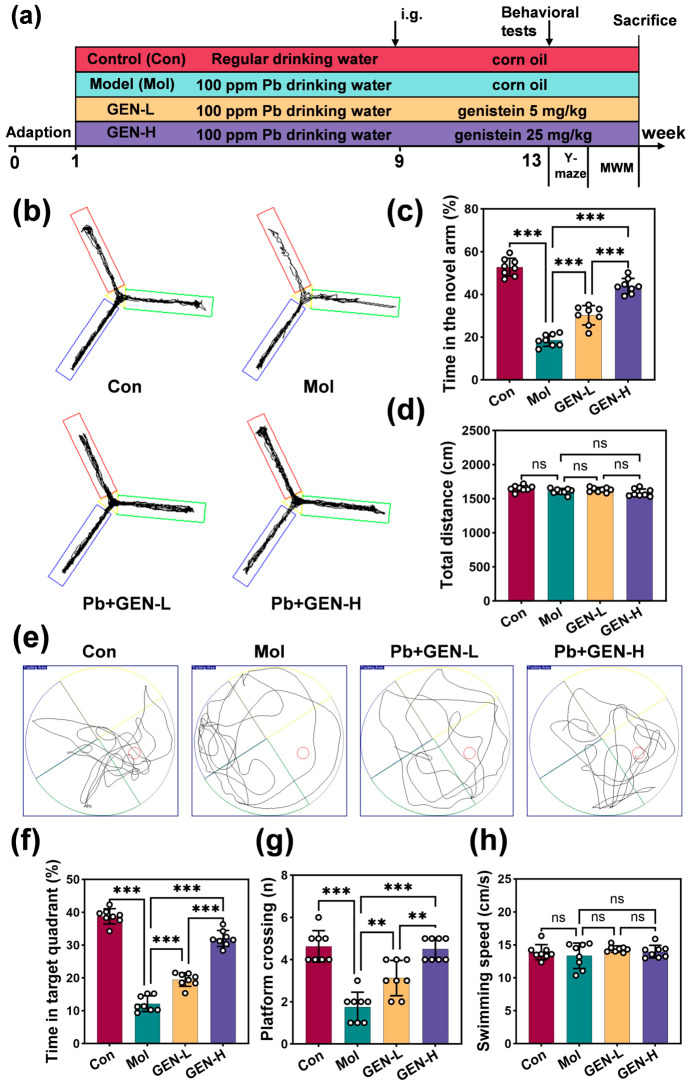
Genistein ameliorates lead-induced learning and memory impairment in mice. (**a**) Experimental flowchart of the study design. (**b**) Representative movement trajectories of mice in the Y-maze test. (**c**) Percentage of time spent in the novel arm during the Y-maze novel arm test. (**d**) Total distance traveled by mice in the Y-maze test. (**e**) Representative swimming trajectories of mice during the probe trial of the Morris water maze. (**f**) Percentage of time spent in the target quadrant during the probe trial. (**g**) Number of platform crossings during the probe trial. (**h**) Average swimming speed of mice in the Morris water maze. Data are expressed as mean ± SD; *n* = 8. ** *p* < 0.01, *** *p* < 0.001; ns, not significant. GEN, Genistein.

As shown in the experimental schedule, mice were exposed to 100 ppm lead in drinking water, followed by oral administration of genistein at low or high doses before behavioral assessment using the Y-maze and Morris water maze tests (Figure 1a). To determine whether genistein could improve lead-induced recognition memory deficits, mice were first subjected to the Y-maze novel arm test. Representative movement traces showed that control mice actively explored all arms, particularly the novel arm, whereas lead-exposed model mice displayed reduced entry and residence within the novel arm, indicating impaired spatial recognition memory (Figure 1b). Quantitative analysis further confirmed that the percentage of time spent in the novel arm was markedly decreased in the Mol group. In contrast, genistein treatment significantly increased novel-arm exploration in lead-exposed mice, with the high-dose genistein group showing a more pronounced recovery than the low-dose group (Figure 1c). Notably, the total distance traveled in the Y-maze was not significantly different among the groups (Figure 1d), suggesting that the reduced novel-arm preference caused by lead exposure was not due to impaired spontaneous locomotor activity or general exploratory ability. Direct comparison further showed that Pb + GEN-H mice spent significantly more time in the novel arm than Pb + GEN-L mice, whereas total distance remained unchanged among all groups (Figure 1c,d).

To further evaluate spatial learning and memory retention, the Morris water maze probe trial was performed. During the probe test, control mice showed concentrated swimming trajectories in the target quadrant where the platform had previously been located, reflecting intact spatial memory retrieval. In contrast, lead-exposed mice exhibited more dispersed swimming paths and spent less time searching in the target quadrant, indicating impaired memory retention after lead exposure (Figure 1e,f). Consistently, the number of crossings over the former platform location was reduced in the Mol group, further supporting the presence of spatial memory deficits (Figure 1g). Genistein administration markedly improved these lead-induced behavioral abnormalities, as evidenced by increased residence time in the target quadrant and more frequent platform-site crossings. Among the two treatment groups, high-dose genistein produced a stronger improvement in both indices, suggesting a dose-related protective tendency against lead-induced spatial memory impairment (Figure 1f,g). Importantly, the average swimming speed remained comparable among all experimental groups (Figure 1h), indicating that the differences observed in the Morris water maze were unlikely to result from changes in swimming ability, motivation, or motor coordination. Collectively, these behavioral results demonstrate that chronic lead exposure impairs both spatial recognition memory and spatial reference memory in mice. Genistein treatment effectively alleviates these cognitive deficits, particularly at the higher dose, without significantly altering basal locomotor activity or swimming performance. These findings suggest that genistein exerts a protective effect against lead-induced learning and memory impairment. Similarly, Pb + GEN-H mice showed significantly greater target-quadrant residence time and platform crossings than Pb + GEN-L mice, while swimming speed did not differ among groups (Figure 1f–h).

### 2.2. Genistein Attenuates Lead-Induced Hippocampal Injury and Neuronal Apoptosis

**Figure 2:**
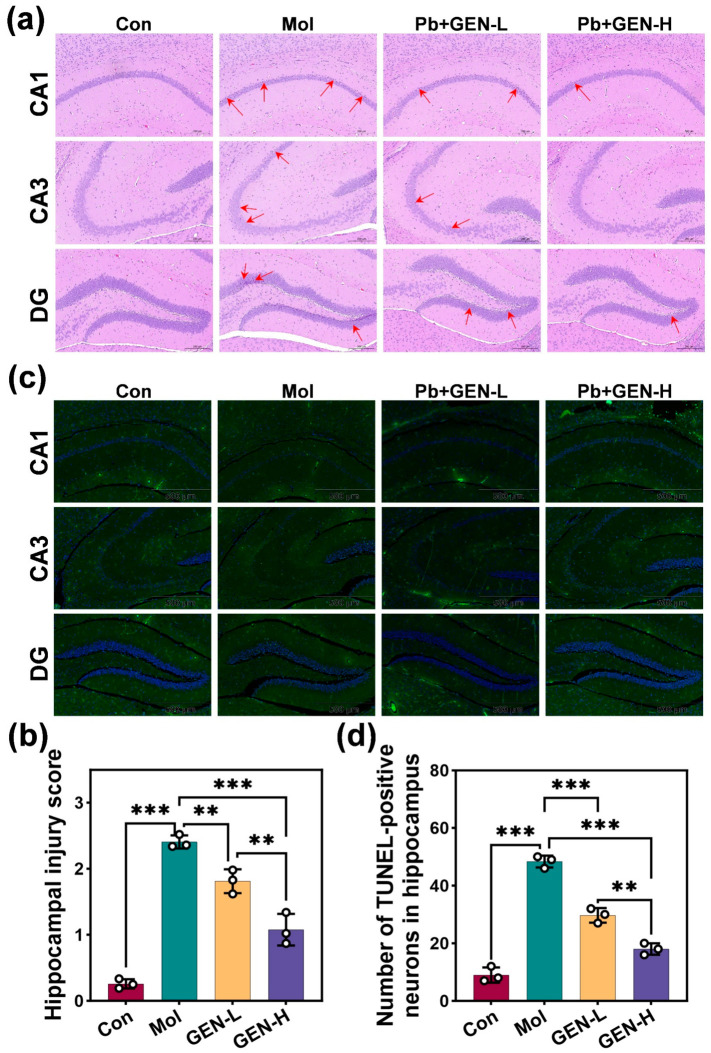
Effects of genistein on hippocampal histopathological injury and apoptosis in lead-exposed mice. (**a**) Representative H&E staining images of the CA1, CA3, and DG regions of the hippocampus from the indicated groups. Red arrows indicate abnormal neuronal morphology or histopathological alterations. Scale bar = 200 μm. (**b**) Quantitative analysis of hippocampal injury scores in each group. (**c**) Representative TUNEL staining images of the CA1, CA3, and DG regions of the hippocampus from the indicated groups. Green fluorescence indicates TUNEL-positive cells, and blue fluorescence indicates DAPI-stained nuclei. Scale bar = 500 μm. (**d**) Quantitative analysis of the number of TUNEL-positive neurons in the hippocampus. Data are presented as mean ± SD. *n* = 3 biologically independent animals per group. ** *p* < 0.01, *** *p* < 0.001.

To investigate whether the cognitive improvement induced by genistein was accompanied by histological protection in the hippocampus, hematoxylin and eosin (H&E) staining was performed in three major hippocampal subregions, including CA1, CA3, and DG. As shown in Figure 2a, hippocampal neurons in the control group exhibited relatively compact and orderly arrangements, with well-preserved cellular morphology and clear regional organization. In contrast, mice exposed to lead displayed obvious histopathological abnormalities across the examined hippocampal areas. In the CA1 and CA3 regions, lead exposure resulted in disrupted neuronal alignment, loosened cell arrangement, and increased numbers of morphologically abnormal neurons. Similar pathological changes were also observed in the DG region, where the granule cell layer appeared less organized and neuronal morphology was visibly impaired. These alterations indicate that chronic lead exposure caused structural damage to hippocampal neuronal populations that are closely associated with learning and memory function.

Genistein administration alleviated the lead-induced histopathological alterations in the hippocampus. Compared with the Mol group, mice treated with genistein showed improved neuronal organization and more intact hippocampal architecture in the CA1, CA3, and DG regions. The low-dose genistein group exhibited partial attenuation of lead-induced morphological damage, whereas the high-dose genistein group showed a more evident preservation of neuronal arrangement and tissue integrity. These observations suggest that genistein, particularly at the higher dose, mitigates lead-induced hippocampal structural injury. Blinded histopathological scoring further confirmed that lead exposure markedly increased hippocampal injury scores, and genistein significantly reduced these scores, with Pb + GEN-H showing a greater reduction than Pb + GEN-L (Figure 2b).

To further assess whether lead exposure induced neuronal apoptosis in the hippocampus, TUNEL staining was performed. As shown in Figure 2c, only weak TUNEL-positive signals were observed in the hippocampal regions of control mice, indicating a relatively low level of apoptotic cell death under normal conditions. In contrast, lead-exposed mice showed markedly enhanced green fluorescence in the CA1, CA3, and DG regions, suggesting that lead exposure promoted apoptosis-like DNA fragmentation in hippocampal cells. After genistein treatment, the intensity and distribution of TUNEL-positive signals were visibly reduced compared with the Mol group. This reduction was more apparent in the high-dose genistein group, indicating that genistein attenuated lead-induced hippocampal apoptosis in a dose-related manner. Quantification of TUNEL-positive neurons further confirmed that lead exposure increased hippocampal apoptosis, whereas genistein reduced the number of TUNEL-positive neurons, with a stronger effect in the Pb + GEN-H group than in the Pb + GEN-L group (Figure 2d).

### 2.3. Genistein Reduces Lead Burden and Improves Hippocampal Redox Balance in Lead-Exposed Mice

**Figure 3:**
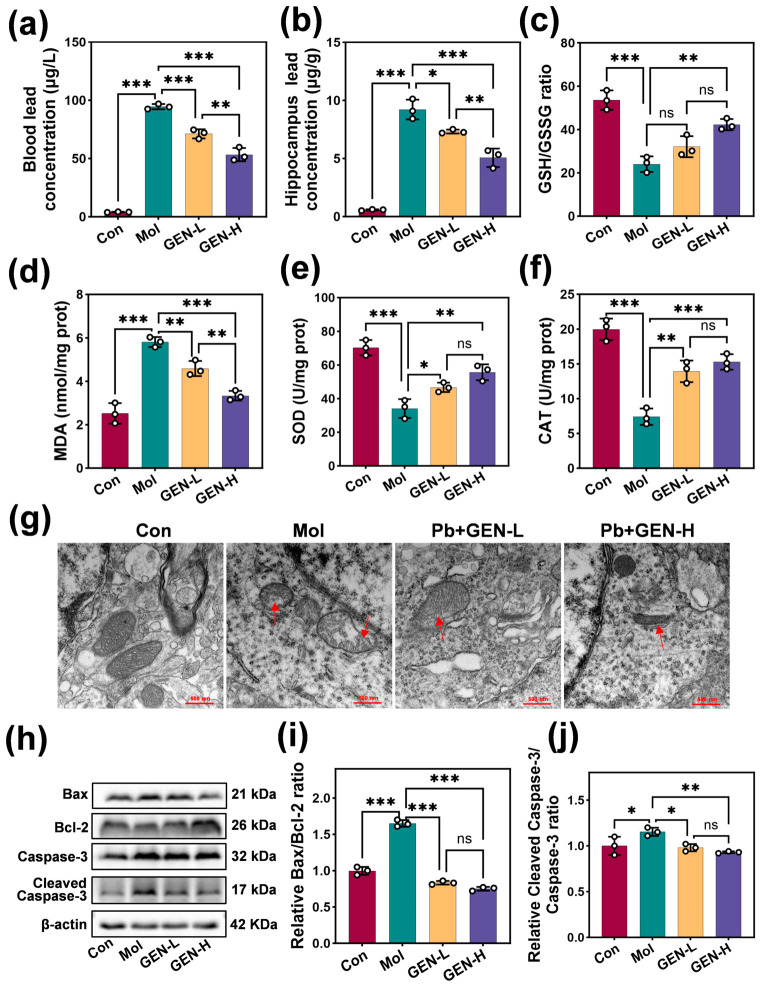
Effects of genistein on lead accumulation, oxidative stress, mitochondrial ultrastructure, and apoptosis-related protein expression in lead-exposed mice. (**a**) Blood lead concentration in mice from each group. (**b**) Lead concentration in hippocampal tissues. (**c**) GSH/GSSG ratio in hippocampal tissues. (**d**–**f**) Hippocampal levels of MDA, SOD activity, and CAT activity. (**g**) Representative transmission electron microscopy images showing mitochondrial ultrastructure in hippocampal tissues from the indicated groups. Red arrows indicate representative abnormal mitochondria. Scale bar = 500 nm. (**h**) Representative immunoblots of Bax, Bcl-2, Caspase-3, and Cleaved Caspase-3 in hippocampal tissues. (**i**) Quantitative analysis of the Bax/Bcl-2 ratio. (**j**) Quantitative analysis of the Cleaved Caspase-3/Caspase-3 ratio. Data are expressed as mean ± SD, *n* = 3 biologically independent animals per group. The three animals were randomly selected from the eight animals in each group after behavioral testing and before tissue-based measurements, without reference to behavioral, biochemical, histological, or molecular outcomes. * *p* < 0.05, ** *p* < 0.01, *** *p* < 0.001; ns, not significant. MDA, Malondialdehyde; SOD, superoxide dismutase; CAT, catalase; GSH/GSSG, glutathione/oxidized glutathione.

To determine whether the protective effect of genistein was associated with changes in lead accumulation, lead concentrations in blood and hippocampal tissues were measured. As shown in Figure 3a,b, mice in the Mol group exhibited a pronounced increase in blood and hippocampal lead levels compared with the control group, confirming successful systemic lead exposure and hippocampal lead deposition. Genistein treatment significantly decreased lead concentrations in both blood and hippocampal tissues. Notably, the reduction was more evident in the high-dose genistein group, suggesting that genistein intervention attenuated lead-associated body burden and limited hippocampal lead accumulation in a dose-related manner. These findings indicate that reduced internal lead burden, particularly reduced hippocampal lead accumulation, may represent one important contributor to the neuroprotective phenotype observed after genistein treatment. Direct comparison between the two genistein doses confirmed significantly lower blood and hippocampal lead concentrations in the Pb + GEN-H group than in the Pb + GEN-L group (Figure 3a,b).

Because lead accumulation is closely associated with oxidative injury, hippocampal redox status was further evaluated. The GSH/GSSG ratio, an important indicator of intracellular redox balance, was markedly reduced in the Mol group, indicating that lead exposure disturbed the glutathione antioxidant system in hippocampal tissues. Low-dose genistein produced a moderate increase in the GSH/GSSG ratio, although this change did not reach statistical significance, whereas high-dose genistein significantly restored the GSH/GSSG ratio compared with the Mol group (Figure 3c). These results suggest that genistein, particularly at the high dose, improves the lead-induced disruption of hippocampal glutathione homeostasis. However, the difference between Pb + GEN-L and Pb + GEN-H did not reach statistical significance for the GSH/GSSG ratio (Figure 3c).

Consistent with impaired antioxidant defense, lead exposure markedly increased hippocampal malondialdehyde (MDA) levels, reflecting enhanced lipid peroxidation. In parallel, the activities of two major antioxidant enzymes, superoxide dismutase (SOD) and catalase (CAT), were substantially decreased in the Mol group (Figure 3d–f). Genistein administration significantly reversed these oxidative stress-related alterations. Both low- and high-dose genistein reduced MDA accumulation and enhanced SOD and CAT activities, although the difference between the two genistein doses reached significance for MDA but not for SOD or CAT. Collectively, these data indicate that genistein alleviates lead-induced oxidative damage in the hippocampus, which is accompanied by reducing lipid peroxidation and improving endogenous antioxidant capacity.

### 2.4. Genistein Preserves Mitochondrial Ultrastructure and Suppresses Hippocampal Apoptotic Signaling

Given that oxidative imbalance may be accompanied by mitochondrial injury, the mitochondrial ultrastructure in hippocampal tissues was examined by transmission electron microscopy. In the control group, mitochondria displayed relatively normal morphology, with intact membranes and clearly organized cristae (Figure 3g). In contrast, hippocampal mitochondria from lead-exposed mice showed obvious ultrastructural abnormalities, including mitochondrial swelling, disrupted or blurred cristae, and impaired structural integrity. These morphological changes indicate that lead exposure induced mitochondrial damage in hippocampal cells.

Genistein treatment visibly ameliorated lead-induced mitochondrial abnormalities. Compared with the Mol group, mitochondria in the genistein-treated groups exhibited more preserved morphology and relatively clearer cristae structures. The protective effect was especially apparent in the high-dose genistein group, in which mitochondrial swelling and cristae disruption were less severe. These observations suggest that genistein helps maintain mitochondrial integrity in the hippocampus under lead-exposure conditions.

To further assess whether mitochondrial protection was accompanied by reduced apoptotic signaling, apoptosis-related proteins were analyzed by Western blotting. Lead exposure markedly increased the Bax/Bcl-2 ratio, indicating a shift toward a pro-apoptotic state in hippocampal tissues (Figure 3h,i). In addition, the Cleaved Caspase-3/Caspase-3 ratio was elevated in the Mol group, suggesting activation of the caspase-dependent apoptotic pathway (Figure 3h–j). Genistein administration significantly decreased both the Bax/Bcl-2 ratio and the Cleaved Caspase-3/Caspase-3 ratio compared with the Mol group. However, direct comparison between Pb + GEN-L and Pb + GEN-H did not show significant differences in these apoptosis-related protein ratios.

### 2.5. Metabolomic Profiling Associates Genistein-Mediated Neuroprotection with Glutathione-Centered Redox Metabolism

**Figure 4:**
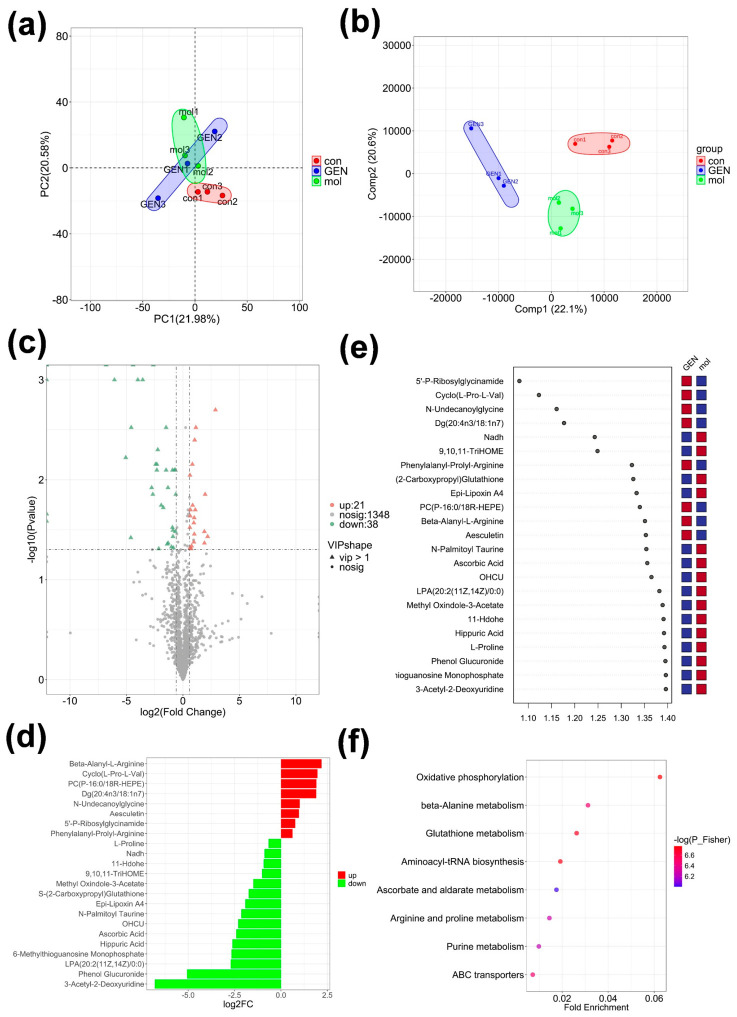
Untargeted metabolomic analysis of brain tissues from lead-exposed mice following genistein treatment. (**a**) PCA score plot of the metabolic profiles in brain tissues from each group. (**b**) PLS-DA score plot. (**c**) Volcano plot of differential metabolites between the GEN and Mol groups. (**d**) Differential metabolites ranked by log2FC; red indicates upregulation and green indicates downregulation. (**e**) Major differential metabolites selected based on VIP values; gray dashed lines are used as visual reference guides, and the adjacent color blocks indicate relative abundance in the GEN and Mol groups, with red and blue representing relatively higher and lower abundance, respectively. (**f**) KEGG pathway enrichment analysis. Con, control group; Mol, lead-exposed model group; GEN, lead exposure plus genistein (25 mg/kg) treatment group. *n* = 3.

To further explore the metabolic basis underlying the neuroprotective effect of genistein, untargeted metabolomic profiling was performed using whole-brain tissue samples. PCA revealed a tendency toward metabolic separation among the control, model, and genistein-treated groups, suggesting that lead exposure disturbed the brain metabolic profile and that genistein treatment induced metabolic remodeling (Figure 4a). PLS-DA analysis further showed clearer separation among the three groups, indicating that genistein markedly altered the metabolic pattern of lead-exposed mice (Figure 4b).

Differential metabolite analysis between the GEN and model groups identified multiple significantly altered metabolites, including 21 upregulated and 38 downregulated metabolites (Figure 4c). Among the major differential metabolites ranked by log2FC and VIP values, several metabolites related to redox regulation and energy metabolism were identified, including 5′-P-ribosylglycinamide, N-undecanoylglycine, NADH, ascorbic acid, and S-(2-carboxypropyl) glutathione (Figure 4d,e). KEGG analysis suggested that these metabolites were mainly enriched in oxidative phosphorylation and glutathione-related pathways (Figure 4f). Notably, the enrichment of glutathione metabolism was consistent with the restoration of the hippocampal GSH/GSSG ratio observed after genistein treatment.

**Figure 5:**
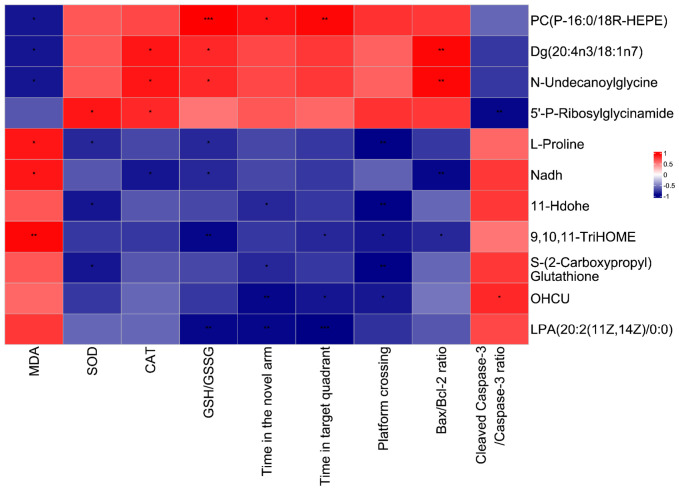
Correlation analysis between differential brain metabolites and oxidative stress-, behavior-, and apoptosis-related indices. * *p* < 0.05, ** *p* < 0.01, *** *p* < 0.001.

Spearman’s correlation analysis was used to assess associations between key metabolites and indicators of oxidative stress, behavioral changes, and apoptosis. The correlation heatmap showed that several metabolites were closely associated with MDA, SOD, CAT, GSH/GSSG ratio, spatial memory parameters, Bax/Bcl-2 ratio, and Cleaved Caspase-3/Caspase-3 ratio (Figure 5). These unbiased metabolomic and correlation analyses suggest an association between genistein-mediated neuroprotection to glutathione-centered redox remodeling.

### 2.6. Inhibition of Glutathione Synthesis Compromises the Antioxidant and Anti-Apoptotic Effects of Genistein in Lead-Exposed HT22 Cells

**Figure 6:**
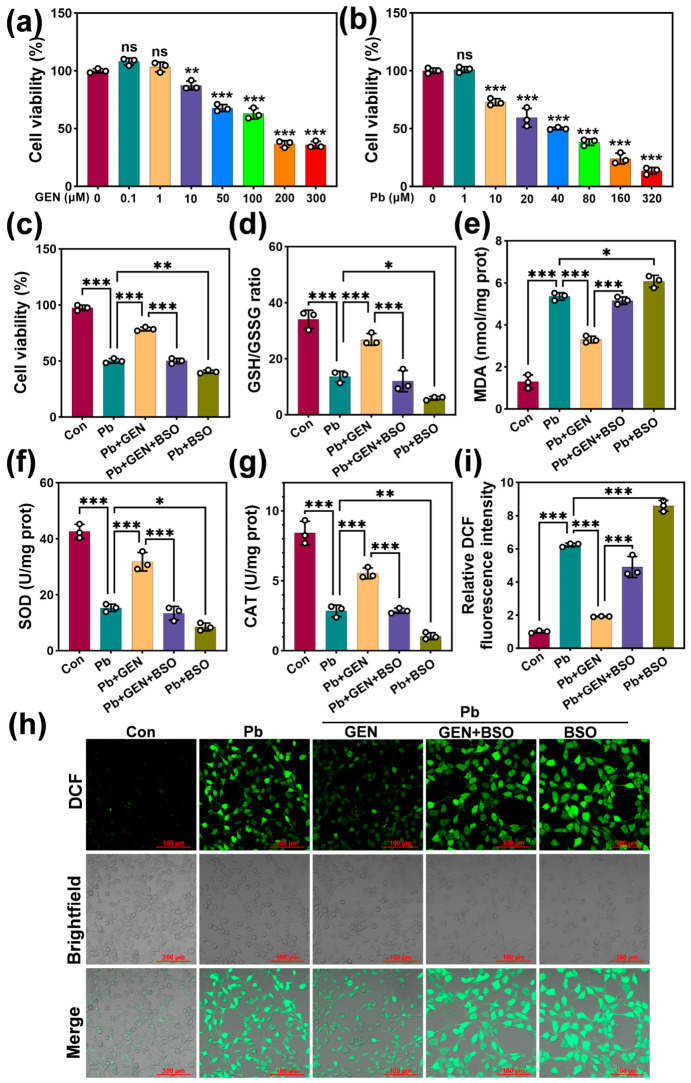
Genistein attenuates lead-induced oxidative injury in HT22 cells. (**a**,**b**) Effects of different concentrations of genistein and lead on HT22 cell viability. (**c**) Cell viability of HT22 cells subjected to the indicated treatments. For panels (**c**–**i**), cells were treated with 40 μM lead and/or 1 μM genistein, and BSO was used at 7.5 μM where indicated. (**d**) Intracellular GSH/GSSG ratio in each group. (**e**–**g**) MDA level, SOD activity, and CAT activity in HT22 cells. (**h**) Representative fluorescence images of intracellular ROS; green fluorescence indicates DCF-detected ROS signals. Scale bar = 100 μm. (**i**) Quantification of relative DCF fluorescence intensity. BSO was used as an inhibitor of glutathione synthesis. Data are expressed as mean ± SD, *n* = 3 independent biological replicates; technical wells or image fields were averaged within each independent experiment. * *p* < 0.05, ** *p* < 0.01, *** *p* < 0.001; ns, not significant. BSO, buthionine sulfoximine.

To further verify whether glutathione homeostasis is involved in the protective effect of genistein, HT22 cells, an immortalized mouse hippocampal neuronal cell line, were treated with lead and genistein in the presence or absence of buthionine sulfoximine (BSO), an inhibitor of glutathione synthesis. Cell viability assays showed that low concentrations of genistein had no obvious cytotoxicity, whereas lead exposure reduced HT22 cell viability (Figure 6a,b). Genistein significantly improved the viability of lead-exposed HT22 cells, while BSO co-treatment markedly weakened this protective effect (Figure 6c).

Consistent with the in vivo findings, lead exposure decreased the intracellular GSH/GSSG ratio, increased MDA levels, reduced SOD and CAT activities, and enhanced DCF fluorescence intensity, indicating severe oxidative injury. Genistein treatment restored the GSH/GSSG ratio, reduced lipid peroxidation and ROS accumulation, and improved antioxidant enzyme activities. However, these antioxidant effects were largely compromised by BSO co-treatment (Figure 6d–i).

**Figure 7:**
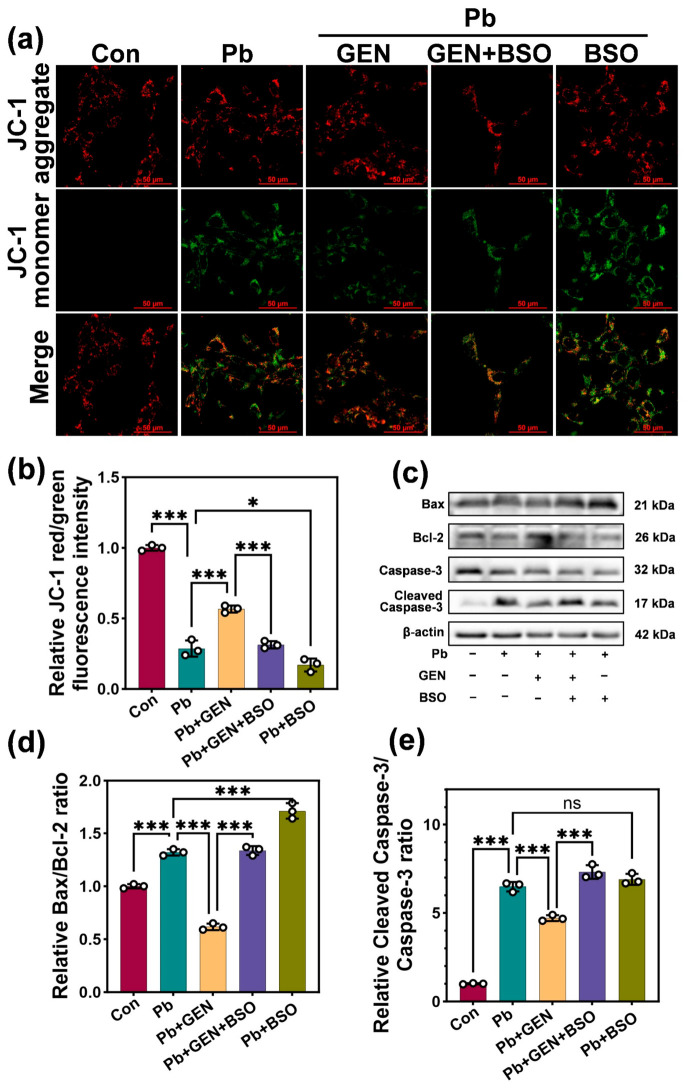
Effects of genistein on lead-induced mitochondrial membrane potential disruption and apoptosis-related protein expression in HT22 cells. (**a**) Representative fluorescence images of mitochondrial membrane potential assessed by JC-1 staining in HT22 cells from the indicated groups. Red fluorescence indicates JC-1 aggregates, and green fluorescence indicates JC-1 monomers. Scale bar = 50 μm. (**b**) Quantification of the JC-1 red/green fluorescence intensity ratio. (**c**) Representative immunoblots of Bax, Bcl-2, Caspase-3, and Cleaved Caspase-3 in HT22 cells. (**d**) Quantitative analysis of the Bax/Bcl-2 ratio. (**e**) Quantitative analysis of the Cleaved Caspase-3/Caspase-3 ratio. Cells were treated with 40 μM lead, 1 μM genistein, and/or 7.5 μM BSO as indicated. Data are expressed as mean ± SD, *n* = 3 independent biological replicates. * *p* < 0.05, *** *p* < 0.001; ns, not significant, as indicated.

We next examined mitochondrial function and apoptosis. JC-1 staining showed that lead exposure caused mitochondrial membrane potential collapse, as reflected by a reduced red/green fluorescence ratio. Genistein partially restored mitochondrial membrane potential, whereas BSO significantly attenuated this effect (Figure 7a,b). Following lead treatment, the expression ratio of Bax to Bcl-2 increased, accompanied by a marked elevation in the Cleaved Caspase-3/Caspase-3 ratio, indicating activation of apoptosis. Genistein intervention reduced these apoptotic markers, whereas combined treatment with BSO abolished this protective effect (Figure 7c–e).

Therefore, pharmacological inhibition of glutathione synthesis functionally compromises genistein-mediated protection, supporting a glutathione-dependent redox–mitochondrial apoptosis axis in lead-exposed HT22 cells.

## 3. Discussion

The present work suggests that genistein-mediated protection against lead-induced hippocampal neurotoxicity and cognitive impairment is accompanied by glutathione-related redox remodeling, mitochondrial preservation, and reduced apoptotic signaling. Rather than positioning genistein simply as a general antioxidant, our findings suggest that this dietary isoflavone may act in association with restoration of an endogenous redox-buffering program centered on glutathione homeostasis. This interpretation is supported by the convergence of behavioral, histopathological, biochemical, ultrastructural, metabolomic, and pharmacological evidence. In particular, the metabolomic enrichment of glutathione metabolism and the loss of genistein-mediated protection after BSO-mediated inhibition of glutathione synthesis provide a possible framework linking redox remodeling to mitochondrial preservation and neuronal survival.

The absence of significant differences in Y-maze total distance and Morris water maze swimming speed is important for interpreting the behavioral outcomes. These findings suggest that lead exposure and genistein treatment did not markedly affect general locomotor activity or swimming performance under the present conditions. Therefore, the observed changes in novel-arm exploration, target-quadrant residence time, and platform crossings are more likely to reflect alterations in spatial learning and memory rather than motor dysfunction. Lead-induced cognitive dysfunction is generally considered to arise from the interaction of metal accumulation, oxidative injury, mitochondrial damage, synaptic disturbance, and neuronal death [18,19]. Among these events, oxidative stress is not merely a downstream consequence of lead exposure; it may represent a nodal pathological process that converts metal burden into cellular dysfunction. Lead can disturb antioxidant enzyme activities, impair thiol-dependent buffering systems, and promote lipid peroxidation, thereby placing hippocampal neurons under sustained redox pressure [1,3]. The hippocampus is particularly vulnerable to this form of injury because of its high metabolic demand, dense excitatory circuitry, and dependence on mitochondrial energy supply for plasticity-related processes. Therefore, the restoration of redox homeostasis may be a prerequisite for meaningful recovery of hippocampal function after lead exposure.

An important conceptual implication of our work is that the protective effect of genistein should not be interpreted solely through a direct radical-scavenging model. Although genistein possesses phenolic hydroxyl groups and has been widely described as an antioxidant phytochemical, food-derived polyphenols often exert their biological effects by modulating adaptive cellular defense networks rather than by stoichiometrically neutralizing reactive oxygen species [20,21]. In this context, the recovery of the GSH/GSSG ratio after genistein treatment is particularly informative. The GSH/GSSG couple reflects the intracellular redox environment and determines the capacity of neurons to buffer peroxides, maintain protein thiol status, and resist mitochondrial oxidative injury [22]. Thus, genistein appears to shift the hippocampal environment from a pro-oxidative state toward a more reducing and cytoprotective state.

Mitochondria provide a mechanistic bridge between redox imbalance and neuronal apoptosis [23]. Sustained oxidative stress can damage mitochondrial membranes, destabilize cristae architecture, impair oxidative phosphorylation, and facilitate the activation of the Bcl-2 family-regulated apoptotic cascade [24,25]. From this perspective, the lead-induced increase in the Bax/Bcl-2 ratio and activation of Caspase-3 should not be regarded as isolated apoptotic events, but as manifestations of overall mitochondrial impairment. Genistein-mediated preservation of mitochondrial ultrastructure and suppression of apoptotic signaling therefore support a model in which redox recovery occurs upstream of mitochondrial stabilization. This interpretation is consistent with previous reports showing that genistein can inhibit mitochondria-dependent apoptosis and ROS-driven inflammatory signaling in neuronal injury models [16,26].

The metabolomic data extend this mechanism beyond classical biochemical assays. Pathway enrichment indicated that genistein treatment reshaped metabolic networks related to glutathione metabolism, oxidative phosphorylation, ascorbate and aldarate metabolism, arginine and proline metabolism, and purine metabolism. These pathways should not be viewed as independent findings. Rather, they point to an integrated redox–bioenergetic adaptive response. Glutathione metabolism provides thiol-buffering capacity; ascorbate metabolism contributes to antioxidant recycling; oxidative phosphorylation reflects mitochondrial energy status; purine metabolism is closely related to nucleotide turnover and energy demand; and arginine/proline metabolism may participate in nitrogen balance, mitochondrial substrate utilization, and stress adaptation [27,28,29]. Together, these metabolic changes suggest that genistein does not simply suppress injury signals, but partially restores the metabolic environment required for neuronal survival.

The correlation analysis further strengthens this interpretation by connecting metabolic remodeling with functional and molecular outcomes. Although correlation cannot establish causality, the observed associations among differential metabolites, oxidative stress indices, cognitive parameters, and apoptosis-related markers suggest that the metabolic state of the brain is closely linked to the severity of lead-induced neurotoxicity. This is important because it moves the discussion beyond a single-target mechanism. In environmental toxicology, especially in heavy metal-induced neurotoxicity, injury rarely depends on one linear pathway [30]. Instead, toxicant exposure produces a network-level collapse involving redox buffering, mitochondrial respiration, lipid integrity, and cell-death control [31]. Genistein appears to counteract this collapse by restoring a more coordinated redox–metabolic phenotype.

When glutathione synthesis was inhibited, genistein lost a substantial part of its ability to restore cell viability, reduce ROS and lipid peroxidation, maintain mitochondrial membrane potential, and suppress Bax/Caspase-3-associated apoptosis in lead-exposed HT22 cells. This finding is critical because it distinguishes a merely correlative antioxidant effect from a functionally important mechanism that contributes to the protective response. In other words, the protection afforded by genistein is not fully preserved when the cellular glutathione synthetic capacity is blocked. This supports the conclusion that glutathione-dependent redox regulation is not just a marker of genistein activity but an important contributor to its protective effect in HT22 cells.

This work also advances the existing literature on genistein and lead neurotoxicity. Previous work demonstrated that genistein alleviates lead-induced spatial memory impairment and neuronal apoptosis, partly through Nrf2-dependent antioxidant signaling and MAPK/NF-κB-associated pathways [17]. Our findings are consistent with those observations but add a distinct metabolic and functional layer. By integrating untargeted metabolomics with BSO-based intervention, the present work identifies glutathione-related redox remodeling as one important component associated with genistein-mediated neuroprotection. However, this mechanism should be considered together with the observed reduction in blood and hippocampal lead burden. The decrease in hippocampal lead accumulation may directly reduce target-tissue toxic exposure, whereas glutathione-related redox remodeling may enhance neuronal resistance to residual lead-induced oxidative and mitochondrial stress. This does not exclude Nrf2, NF-κB, MAPK, or estrogen receptor-related mechanisms. Instead, these pathways may converge on the ability of neurons to preserve glutathione availability, mitochondrial competence, and apoptotic resistance. From this perspective, glutathione homeostasis may serve as a biochemical hub through which multiple genistein-sensitive signaling pathways translate into neuroprotection.

The dietary origin of genistein also deserves attention [17]. As a soybean-derived isoflavone, genistein represents a class of food-derived compounds with potential relevance for long-term intervention against low-level environmental toxicant exposure. This is particularly meaningful for lead neurotoxicity, where complete exposure avoidance is often difficult and therapeutic options after chronic exposure remain limited. Several limitations should be acknowledged. First, BSO was used in HT22 cells but not in vivo. Therefore, the glutathione dependence of genistein-mediated neuroprotection is functionally supported in vitro but remains to be directly confirmed in the intact hippocampus. Second, HT22 cells are useful for mechanistic verification but cannot fully reproduce the cellular complexity of the hippocampus, including astrocyte–neuron glutathione exchange, microglial responses, and blood–brain barrier regulation. Third, because this study emphasized direct internal exposure indicators, including blood and hippocampal lead concentrations, drinking-water consumption was not monitored prospectively. Future studies that incorporate water-intake monitoring would further improve estimation of oral lead exposure. Fourth, although genistein reduced blood and hippocampal lead concentrations, the present study did not determine how genistein altered lead toxicokinetics, including intestinal absorption, systemic distribution, blood–brain barrier transport, or excretion. Therefore, we cannot quantify the relative contribution of reduced hippocampal lead burden versus glutathione redox restoration to the overall neuroprotective outcome. Future studies using lead kinetic analysis, matched hippocampal lead-burden models, and in vivo manipulation of glutathione synthesis will be required to distinguish these mechanisms more precisely. In addition, the present study was designed to evaluate genistein-mediated protection rather than to perform a head-to-head comparison with established chelating or antioxidant interventions. Including such reference interventions in future studies would help benchmark the relative efficacy of genistein and clarify the contribution of lead-burden reduction versus redox-based neuroprotection.

## 4. Materials and Methods

### 4.1. Animal Experiments

Thirty-two male SPF C57BL/6J mice aged 6 weeks with body weights ranging from 18 to 22 g were purchased from Changchun Yisi Laboratory Animal Technology Co., Ltd. (Changchun, China). The mice were not genetically modified and had not undergone any previous experimental procedures before enrolment in the present study. The overall experimental design is illustrated in Figure 1a. After one week of acclimatization, the animals were randomly divided into four groups with eight mice per group: control group (Con, *n* = 8), lead-exposed model group (Mol, *n* = 8), low-dose genistein group (GEN-L, *n* = 8), and high-dose genistein group (GEN-H, *n* = 8). Mice in the Mol, GEN-L, and GEN-H groups received drinking water containing 100 ppm lead acetate (Shanghai Yuanye Bio-Technology Co., Ltd., Shanghai, China), whereas mice in the Con group received regular drinking water. The anhydrous lead acetate concentration of 100 ppm was selected based on previously published studies in which this exposure level effectively induced lead accumulation, hippocampal neuronal injury, and cognitive impairment in mice [32]. Genistein (Shanghai Yuanye Bio-Technology Co., Ltd., Shanghai, China) was administered at 5 and 25 mg/kg; 25 mg/kg was selected as a biologically relevant high dose based on prior in vivo evidence of genistein-mediated neuroprotection, whereas 5 mg/kg was used as a lower exploratory dose to evaluate partial protection and dose-related tendency. All animal experimental procedures were approved by the Animal Ethics Committee of Jilin University (Approval No. 2025-326). The statistical analysis plan was finalized before data analysis but was not registered in a public repository. Treatment administration and outcome measurements were performed in a randomized or interleaved order across groups, and cage positions were regularly rotated to minimize potential order and location effects. At the end of the experiment, mice were deeply anesthetized with sodium pentobarbital and euthanized by cervical dislocation. Death was confirmed by the absence of respiration, heartbeat, and pedal reflex before tissue collection. Humane endpoints included more than 20% body-weight loss, persistent inability to eat or drink, severe lethargy, impaired ambulation, labored breathing, or other signs of severe or unrelieved distress. Animals reaching a humane endpoint would be euthanized immediately. No animals reached the predefined humane endpoints, no animals were excluded, and no expected or unexpected adverse events occurred during this study. Group sizes were based on comparable published studies and the principle of minimizing animal use.

### 4.2. Behavioral Tests

#### 4.2.1. Y-Maze Test

The Y-maze apparatus consisted of three identical arms designated as the start arm, familiar arm, and novel arm. During the acquisition phase, the novel arm was closed, and each mouse was allowed to freely explore start arm and familiar arm for 5 min. After a 1 h intertrial interval, the novel arm was opened for the test phase, and the mouse was placed back into the maze and allowed to explore all three arms for 5 min.

#### 4.2.2. Morris Water Maze Test

Spatial memory was evaluated by Morris water maze. Mice were trained in a 1.6 m pool (22 ± 1 °C) with a platform in quadrant I (visible on day 1, hidden on days 2–6). Four 60-s trials were given daily. On day 7, probe trial was performed to measure target quadrant time, platform crossings and swimming speed [33]. The percentage of time spent in the target quadrant during the probe trial was prespecified as the primary outcome measure.

### 4.3. Histopathological and TUNEL Staining

After euthanasia, brain tissues were rapidly collected and fixed in 4% paraformaldehyde. The tissues were then embedded in paraffin and cut into 5-μm coronal sections. For histopathological evaluation, sections were subjected to H&E staining to assess morphological changes in the hippocampus. Hippocampal histopathological injury was assessed using a semi-quantitative 4-point scoring system by an investigator blinded to the experimental groups. The CA1, CA3, and DG regions were evaluated based on neuronal arrangement, neuronal morphology, nuclear pyknosis, vacuolation, and apparent neuronal loss. The scoring criteria were as follows: 0, normal hippocampal architecture with orderly neuronal arrangement; 1, mild injury with slight neuronal disorganization or occasional abnormal neurons; 2, moderate injury with obvious neuronal disorganization, increased pyknotic or shrunken neurons, and partial structural disruption; and 3, severe injury with marked neuronal degeneration, vacuolation, or apparent neuronal loss. Scores from the CA1, CA3, and DG regions were averaged to obtain the hippocampal injury score for each animal. Neuronal apoptosis was detected using TUNEL staining according to the manufacturer’s instructions. Nuclei were counterstained with DAPI, and fluorescence images were captured to evaluate TUNEL-positive cells in hippocampal regions.

### 4.4. Determination of Lead Concentrations

At the end of the behavioral experiments, mice were deeply anesthetized, and blood samples were collected by retro-orbital bleeding under deep anesthesia. Whole blood was collected into metal-free EDTA-containing anticoagulant tubes, gently inverted to ensure complete mixing, and temporarily kept on ice. After blood collection, mice were euthanized, and death was confirmed before tissue collection. Blood samples were aliquoted and stored at −80 °C until lead concentration analysis. Blood and hippocampal lead concentrations were determined by inductively coupled plasma mass spectrometry (ICP-MS). For blood lead analysis, whole-blood samples were used rather than serum or plasma. Briefly, whole-blood samples were digested with nitric acid and then diluted to an appropriate volume with ultrapure water before ICP-MS measurement. Lead concentrations were quantified using external calibration standards, and blank samples were included during analysis to monitor background contamination. Hippocampal tissues were weighed, digested with nitric acid, and diluted to an appropriate volume before ICP-MS analysis.

### 4.5. Measurement of Oxidative Stress-Related Indicators

Hippocampal tissues or HT22 cells (Servicebio Technology, Wuhan, China) were homogenized or lysed in ice-cold lysis buffer and centrifuged to collect the supernatant. The GSH/GSSG ratio, MDA level, SOD activity, and CAT activity were measured using commercial assay kits (Nanjing Jiancheng Bioengineering Institute, Nanjing, China). Briefly, the GSH/GSSG ratio was calculated based on the relative contents of reduced and oxidized glutathione. MDA was measured as an index of lipid peroxidation using the thiobarbituric acid-reactive substance method. SOD activity was assessed according to its ability to inhibit superoxide-mediated chromogenic reactions, whereas CAT activity was determined by monitoring the decomposition of hydrogen peroxide.

### 4.6. Transmission Electron Microscopy

Hippocampal specimens were prepared for transmission electron microscopy following routine protocols. In brief, the tissues were initially fixed in glutaraldehyde, followed by secondary fixation with osmium tetroxide. The samples were then dehydrated, embedded in resin, and cut into ultrathin sections. After contrast staining with uranyl acetate and lead citrate, the mitochondrial ultrastructure was observed using TEM.

### 4.7. Untargeted Metabolomic Analysis

Untargeted LC-MS/MS metabolomic analysis was performed using brain tissue samples from the Con, Mol, and GEN-H groups. Three biologically independent samples were analyzed per group, resulting in a total of nine samples. Each sample was obtained from an individual mouse and analyzed separately; no pooled biological samples were used for the metabolomic analysis. Samples were randomized during LC-MS/MS acquisition. Whole-brain tissue was used to obtain an exploratory overview of brain-level metabolic remodeling because hippocampal tissues were primarily allocated to region-focused analyses, including lead concentration, GSH/GSSG ratio, oxidative stress markers, mitochondrial ultrastructure, and apoptosis-related protein expression; therefore, the metabolomic data should be interpreted as global brain metabolic alterations rather than hippocampus-specific changes.

After peak detection, alignment, normalization, and data filtering, multivariate analyses were performed to evaluate the overall metabolic differences among groups. PCA was used as an unsupervised method to visualize global metabolic variation. PLS-DA was used as a supervised exploratory model, and its performance was evaluated using cross-validation and permutation testing. The model parameters, including R2X(cum), R2Y(cum), Q2(cum), and cross-validated accuracy, were recorded. To evaluate potential overfitting, a 200-time permutation test was performed, and the original model was compared with randomly permuted class labels.

For differential metabolite screening between the GEN-H and Mol groups, univariate *p* values were calculated using the Wilcoxon rank-sum test and then adjusted for multiple testing using the Benjamini–Hochberg false discovery rate (FDR) method. Metabolites were considered differential when they met the following criteria: VIP > 1.0, |log2 fold change| > log2(1.2), and FDR-adjusted q value < 0.05. KEGG pathway enrichment analysis was subsequently performed based on the FDR-filtered differential metabolites.

Because of the limited number of biological replicates, PLS-DA was interpreted as an exploratory visualization tool rather than as independent evidence of group separation. The biological interpretation of metabolomic findings was based on FDR-controlled differential metabolite analysis and was further considered together with biochemical and functional validation results.

### 4.8. Cell Culture and Treatments

HT22 cells were maintained in DMEM supplemented with fetal bovine serum and antibiotics under standard culture conditions. To determine appropriate treatment concentrations, cells were exposed to different concentrations of genistein or lead, and cell viability was measured. For mechanistic experiments, cells were divided into Con, Pb, Pb + GEN, Pb + GEN + BSO, Pb + BSO. Cells were exposed to 40 μM lead for 24 h to establish the injury model, followed by treatment with 1 μM genistein for 24 h. For BSO intervention, cells were treated with 7.5 μM BSO for 6 h before subsequent analyses [34]. The 40 μM lead concentration was selected because it produced a reproducible moderate decrease in cell viability without excessive cytotoxicity. The 1 μM genistein concentration showed no obvious intrinsic cytotoxicity and was suitable for testing protection against lead-induced injury. BSO was used at 7.5 μM based on previous work to inhibit glutathione synthesis while minimizing nonspecific cytotoxicity.

### 4.9. Cell Viability Assay

Cell viability of HT22 hippocampal neurons was determined using the Cell Counting Kit-8 (CCK-8) assay. Exponentially growing HT22 cells were seeded into 96-well culture plates at a density of 5 × 10^3^ cells per well and incubated for 24 h to allow for complete cell attachment prior to the indicated drug treatments for an additional 24 h. Following treatment, 10 μL of CCK-8 working solution was added to each well, and the plates were incubated at 37 °C in the dark for 2 h. The absorbance at 450 nm was subsequently measured using a microplate reader. Cell viability was calculated and presented as a percentage relative to the untreated control group.

### 4.10. Detection of Intracellular ROS and Mitochondrial Membrane Potential

Intracellular reactive oxygen species and mitochondrial membrane potential in HT22 cells were detected using DCFH-DA and JC-1 staining kits, which were purchased from Solarbio (Beijing, China). All procedures were performed strictly according to the manufacturers’ instructions. For ROS detection, cells were incubated with DCFH-DA, which is deacetylated intracellularly and oxidized by ROS to generate fluorescent DCF signals. Fluorescence intensity was used to indicate relative intracellular ROS accumulation. For mitochondrial membrane potential analysis, cells were stained with JC-1 dye. Healthy mitochondria promote JC-1 aggregate formation with red fluorescence, whereas mitochondrial depolarization increases JC-1 monomers with green fluorescence. Mitochondrial membrane potential was evaluated using the red/green fluorescence ratio.

### 4.11. Western Blot Analysis

Hippocampal tissues or HT22 cells were lysed in RIPA buffer containing protease inhibitors. Equal amounts of protein were separated by SDS-PAGE and transferred onto PVDF membranes. Membranes were blocked with a rapid blocking buffer and then incubated overnight at 4 °C with the corresponding primary antibodies (Table S1). After incubation with appropriate secondary antibodies, protein bands were visualized using an enhanced chemiluminescence detection system. Band intensities were quantified using ImageJ software 1.54p (National Institutes of Health, Bethesda, MD, USA).

### 4.12. Statistical Analysis

Data are presented as mean ± SD. Statistical analysis was performed using GraphPad Prism 10.1.2 (GraphPad Software, Boston, MA, USA). Normality and homogeneity of variance were assessed using the Shapiro–Wilk test and Levene’s test, respectively. Given the relatively small sample sizes and deviations from normality in some datasets, comparisons among three or more independent groups were performed using the non-parametric Kruskal–Wallis H test followed by Dunn’s multiple-comparison test. Spearman’s correlation analysis was used for metabolite–phenotype associations. A value of *p* < 0.05 was considered statistically significant.

## 5. Conclusions

This work suggests that genistein protects against lead-induced hippocampal injury through effects associated with reduced hippocampal lead accumulation and improved glutathione-related redox homeostasis, thereby preserving mitochondrial integrity and suppressing apoptosis. The integration of behavioral outcomes, hippocampal pathology, redox biochemistry, metabolomics, and BSO-based functional validation supports a model in which glutathione-centered redox remodeling is associated with mitochondrial resilience and cognitive protection. These findings provide a scientifically grounded rationale for further investigation of genistein and related dietary isoflavones as mechanistically defined interventions against environmental pollutant-induced neurotoxicity.

## Data Availability

Data will be made available on request.

## Supplementary Materials

The following supporting information can be downloaded at: https://www.mdpi.com/article/10.3390/molecules31132251/s1, Table S1: Primary antibodies used for Western blot.
