# Supplementary material for: Genistein Protects Against Lead-Induced Cognitive Impairment Through a Glutathione-Dependent Redox–Mitochondrial Apoptosis Axis
Source: Molecules. 2026 Jun 26;31(13):2251. doi: 10.3390/molecules31132251 (PMC13363325; doi:10.3390/molecules31132251)
Supplement: Supplementary file 1 [file molecules-31-02251-s001.zip › molecules-4380941-supplementary.pdf]

Supplementary Material

Table S1. Primary antibodies used for Western blot.

Primary antibody	Manufacturer	Molecular weight (kDa)	Dilution
Bax	Nature Biosciences, Hangzhou, China	21	1:2000
Bcl-2	Nature Biosciences, Hangzhou, China	26	1:2000
Cleaved Caspase-3	Abcam, Cambridge, UK	17	1:1000
Caspase-3	Abcam, Cambridge, UK	32	1:1000
β-Actin	Proteintech, Wuhan, China	42	1:10000
